# How much imitation is there in a shadowing task?

**DOI:** 10.3389/fpsyg.2013.00346

**Published:** 2013-06-21

**Authors:** Sophie Dufour, Noël Nguyen

**Affiliations:** ^1^UMR 7309, Laboratoire Parole et Langage, Aix-Marseille Université, CNRSAix-en-Provence, France; ^2^Brain and Language Research Institute, Aix-Marseille UniversitéAix-en-Provence, France

**Keywords:** shadowing task, phonetic convergence, imitation

## Abstract

Phonetic imitation, also called phonetic convergence, is currently at the heart of numerous investigations since it can inform us on both the nature of lexical representations and the link between production and perception processes in spoken language communication. A task that has been largely used to study phonetic imitation is the shadowing task, in which participants merely listen to and repeat isolated words. In this study, we examined the extent to which the phonetic convergence effect found when participants shadow auditory tokens, is an imitation of the speaker. We thus compared the phonetic convergence effect observed in a shadowing task to that observed when participants were explicitly instructed to imitate the productions they were exposed to. Although the phonetic convergence effect was greater when participants intentionally imitated the speaker's productions, shadowing and imitation instructions led to the same degree of convergence in a post-exposure task. Hence, the convergence effect found in a shadowing task and that found in an imitation task seem to share a general mechanism which is automatic and which taps into the long-term representations of the words in memory. At a more theoretical level, our results reinforce the claim that detailed auditory traces associated with perceived words are stored in memory and are later used for production.

## Introduction

Imitation is an all-pervading process by which individuals adjust to one another in social interaction, and is seen as one of the fundamental mechanisms of human development (Meltzoff et al., 2009). For example, vocal imitation plays an important role in language acquisition, and infants have been found to imitate speech patterns as early as 12 weeks after birth (Kuhl and Meltzoff, 1996). Furthermore, imitation does not stop when language acquisition is completed since it is a recurrent behavior in adults at several linguistic levels. At a low level of processing, increases in similarity in vocal intensity (Natale, 1975) and speech rate (Giles et al., 1991) between talkers have been observed over the course of a conversational exchange. At a higher level of processing, it has been shown that talkers tend to repeat words and grammatical constructions produced by their interlocutors (Branigan et al., 2000; Pickering and Garrod, 2004).

Phonetic imitation, also called phonetic convergence, is the process by which a talker tends to make her/his speech more similar to that of the talker she/he is interacting with. This phenomenon is critical for models that assume a strong link between perception and production processes (Levelt, 1989) as well as for models that postulate that each word in the mental lexicon is associated with many auditory episodes (Goldinger, 1998). In particular, the observation that a talker becomes more similar in her/his production to a target talker as a result of exposure to that talker's speech would indicate that detailed auditory traces associated with perceived words are stored in memory and are later used for production.

In laboratory research, phonetic convergence between speakers has been found both during conversational interactions (Pardo, 2006; Pardo et al., 2010), and in a non-interactional setting, as in the shadowing task, in which participants merely listen to and repeat isolated words (Goldinger, 1998; Namy et al., 2002; Shockley et al., 2004; Babel, 2010, 2012; Babel and Bulatov, 2012). To our knowledge, Goldinger (1998) was the first to report evidence for imitation when participants shadow spoken words. In his study, imitation was assessed by the mean of an AXB task in which participants had to judge which of two stimuli, a baseline stimulus [i.e., stimulus A (B)] recorded by the shadower during a reading task prior to the shadowing task or the shadowed word [i.e., stimulus B (A)] is a better imitation of the token that the shadower heard (i.e., stimulus X). The perceived degree of imitation was systematically affected by word frequency and was higher for rare than for high-frequency words. According to Goldinger (1998), the modulation of the imitation effect as a function of lexical frequency suggests that lexical representations are brought into play in speech imitation. In a follow-up study, Goldinger and Azuma (2004) asked whether imitation toward a target speaker could be observed as a result of mere exposure to that target speaker's voice in a word-identification task. Participants had to read words aloud in two sessions, before and after exposure to training tokens. During training, participants were presented with a series of recorded spoken words and had to retrieve each word within a 40-word grid using a computer mouse. Crucially, participants never spoke the words during training. Perceptual judgments collected during an AXB task revealed that participants' productions after exposure were considered as a better imitation of trained tokens than participants' productions before exposure. These results are particularly interesting since they suggest that lexical representations include detailed traces of the spoken words that a listener is exposed to, which are activated when that listener later reads these words.

Because the AXB task only provides a global perceptual measure of imitation that gives no information about which parameters in the acoustic signal are sensitive to imitation, several studies have recently focused on the acoustic characteristics of imitation. For example, it has been shown that VOT (Shockley et al., 2004) and fundamental frequency (Babel and Bulatov, 2012) are highly imitable phonetic features. In a recent study, Babel (2010) focused on imitation of vowel formant frequencies. She asked New Zealand participants to shadow auditory words pronounced by an Australian English speaker. Although New Zealand participants accommodated their vowels to those of the Australian speaker, they did not converge toward that speaker to the same degree for all vowels. Participants showed more convergence toward the DRESS vowel, a vowel that has a different position in the New Zealand vowel space compared with that of Australian English, than toward the other vowels (e.g., the KIT vowels) examined in that study. These results thus indicate that at least some phonetic features of a speaker's dialect may be imitated by a speaker of another dialect (see also Delvaux and Soquet, 2007).

To sum up, phonetic convergence effect has been repeatedly observed in a shadowing task, even though participants were not explicitly instructed to imitate the productions they heard. The term “imitation” has often been used (Goldinger, 1998; Shockley et al., 2004; Babel, 2012) to refer to the phonetic convergence effect found in this particular task. However, we know that explicit imitation has more impact than simple repetition on sentence comprehension (Adank et al., 2010), and it could be the case that individuals accommodate to a speaker to a greater extent in an imitation than in a shadowing task. Thus, it is important to establish the extent to which the phonetic convergence effect that is found when participants shadow auditory tokens can be viewed as explicit imitation of the speech signal. At a more theoretical level, the comparison of the degree of convergence effects found in the two tasks allows us to determine whether or not the convergence effect found with shadowing instructions is governed by the same mechanisms as that found with imitation instructions. Pardo (2006) and Pardo et al. (2010) has reported somewhat different results according to whether participants were or were not explicitly instructed to imitate the other talker during the course of a conversation. However, to the best of our knowledge, no study has yet examined the difference between explicitly asked-for imitation vs. simple repetition on phonetic convergence for single auditory words.

In this study, we focused on the phonetic convergence effect found in the shadowing task and we compared it to that found in an imitation task in which participants were explicitly instructed to imitate the productions they were exposed to. In particular, we examined phonetic convergence in a cross dialectal experiment in which Southern French participants had to imitate or shadow words pronounced by a Standard French speaker. Contrary to Southern French speakers, Standard French speakers make a contrastive distinction between the /e/ and /ε/ vowels in word final position. The words épée *“sw*ord” and épais “thick” are thus pronounced /epe/ and /epε/, respectively, by Standard French speakers, whereas they are both pronounced /epe/ by Southern French speakers. The experiment involved three phases: a pre-test, test, and post-test phase. During the pre-test, participants read aloud words ending in /e/ and /ε/ in Standard French. This allowed us to establish the participant's baseline productions of the /e/ and /ε/ words. Because in Southern French, the two vowels are pronounced in the same way [i.e., /e/], no difference in *F*_1_ frequency for the final vowel was expected between the /e/ and /ε/ words. During the test phase, participants were presented with words ending in /e/ and /ε/ and recorded by a Standard French speaker. Half of the participants performed a shadowing task and the other half an imitation task. We expected to find a difference in *F*_1_ in the final vowel between the /e/ and /ε/ words as a result of exposure to the Standard French speaker. Moreover, if the phonetic convergence effect generally found in the shadowing task reflects an explicit imitation, the *F*_1_ difference should be of the same magnitude in the shadowing and imitation groups. Finally, in the post-test phase, participants were again asked to perform a reading aloud task. This phase allowed us to examine the persistence of the potential vowel changes resulting from imitation/shadowing on the same task as that used to record the participant's baseline productions.

## Materials and methods

### Participants

Twenty Southern French speakers from Aix-Marseille University took part in the experiment. Half of them were assigned to the shadowing task (eight women, two men, mean age = 26.8), and the other half to the imitation task (eight women, two men, mean age = 26.8). All participants reported having no hearing or speech disorders.

### Materials

Twenty-two bisyllabic words ending in /e/ (e.g., *café* /kafe/ “coffee”) and 22 bisyllabic words ending in /ε/ (e.g., *sachet* /saıntε/ “bag”) were selected from Vocolex, a lexical database of the French language (Dufour et al., 2002). These words were used in the pretest, test, and post-test. Because convergence effects toward the Standard French speaker could be minimized for words that the participant has already pronounced once in her/his own accent during the pre-test, 44 other bisyllabic words, half ending in /e/ and the other half ending in /ε/ in Standard French, were also selected, and were used only in the test and post-test. To divert the participants' attention from the /e/ and /ε/ vowels, 264 filler words that did not contain either of the two critical vowels were also selected. In order for each phase to contain 25% of test words, all of the fillers were used in the test and post-test, and half of them were used in the pre-test phase. For the purposes of the test phase, all of the words were recorded by a female native speaker of Standard French who produces the /e/-/ε/ contrast, in an ane-choic chamber, using high-quality digital recording equipment at a sampling rate of 44100 Hz. The main words' characteristics are given in Table 1, and the individual words are given in Appendix. The label *baseline words* refers to the words used in the pre-test, and the label *new words* refers to the words that were used in the test and post-test only.

**Table 1:** **Characteristics of the words used in the experiment (mean values)**.

	**Baseline words**		**New words**	
	**/e/ vowel**	**/ε/ vowel**	**/e/ vowel**	**/ε/ vowel**
Frequency (in logarithm, base 10)	3.13	3.05	3.23	3.15
Number of syllables	2	2	2	2
Number of phonemes	4.55	4.55	4.55	4.55
Duration (in ms)	572	584	579	581

### Procedure

The experiment took place in the anechoic chamber and participants' productions were recorded using the same equipment as for the Standard French speaker. During the pre- and post-test, words were randomly displayed in lowercase letters in the center of the screen for 2 s. Participants were instructed to read aloud the words as naturally and as clearly as possible. During the test, words were presented auditorily over headphones at a comfortable sound level. Half of the participants were instructed that, upon hearing the word, they were to **repeat it as naturally and as clearly as possible**. The other half were instructed that, upon hearing the word, they were to **repeat it by imitating the speaker's specific pronunciation**.

## Results and discussion

**Figure 1:**
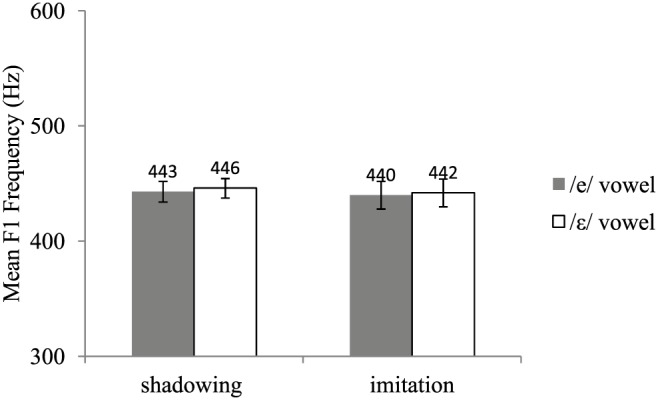
**Mean *F*_1_ frequencies and standard errors for the /e/ and /ε/ vowels in the shadowing and imitation groups for the pre-test**.

**Figure 2:**
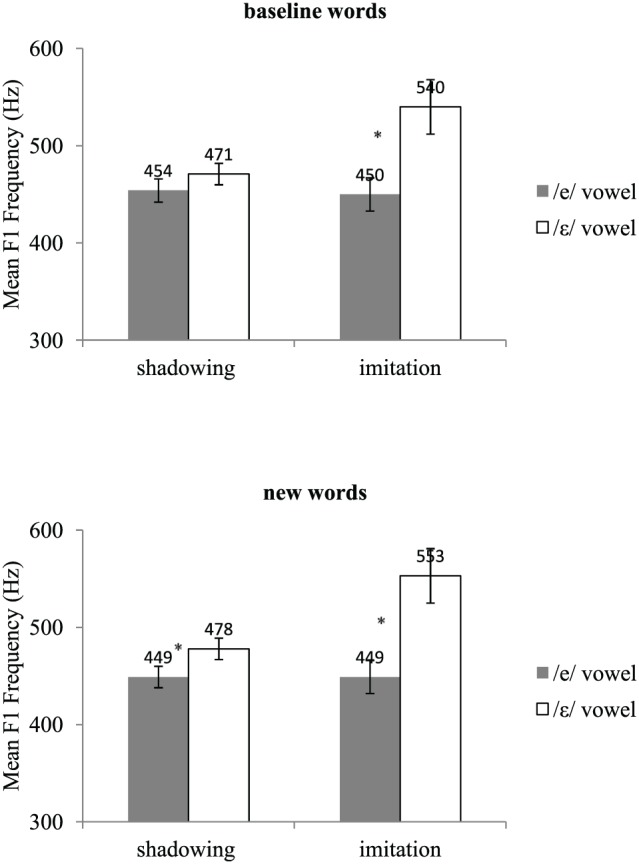
**Mean *F*_1_ frequencies and standard errors for the /e/ and /ε/ vowels in the shadowing and imitation groups for the test**. The label *baseline words* refers to the words used in the pre-test, and the label *new words* refers to the words that were used in the test and post-test only. ^*^lndicates a significant effect of the vowel.

**Figure 3:**
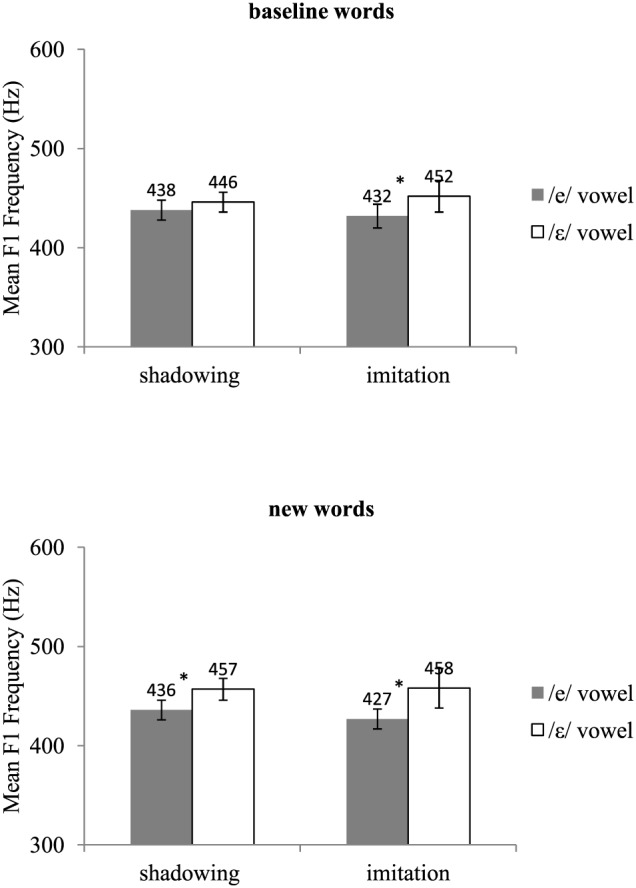
**Mean *F*_1_ frequencies and standard errors for the /e/ and /ε/ vowels in the shadowing and imitation groups for the post-test**. The label *baseline words* refers to the words used in the pre-test, and the label *new words* refers to the words that were used in the test and post-test only. ^*^lndicates a significant difference between the /e/ and /ε/ vowels.

**Figure 4:**
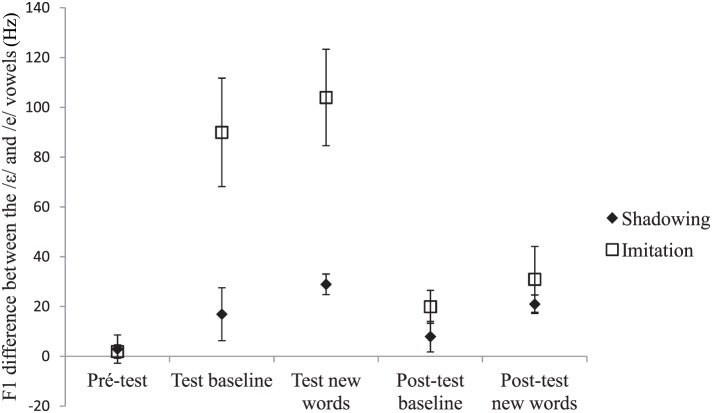
**Average difference in *F*_1_ frequency (Hz) between the /ε/ and /e/ vowels and standard errors for each phase**. The label *baseline words* refers to the words used in the pre-test, and the label *new words* refers to the words that were used in the test and post-test only.

Acoustic recordings were segmented using Praat (Boersma, 2001). For each /e/ and /ε/ word, we located the acoustic onset and offset of the vowel. *F*_1_ frequency was then automatically measured at the vowel's acoustic midpoint using the Burg algorithm as implemented in Praat. Two initial analyses of variance (ANOVAs) by participants, one on the test data and the other on the post-test data, including group (shadowing, imitation), vowel (/e/, /ε/), and type of word (baseline, new words) as variables showed a significant interaction between vowel and type of word in both the test [*F*(1, 18) = 4.14, *p* = 0.05] and post-test phases [*F*(1, 18) = 5.38, *p* < 0.05]. This interaction showed that the difference in *F*_1_ frequency between the /e/ and the /ε/ vowels was slightly but significantly greater for the words not pronounced during the pre-test (new words) than for those included in the pre-test (baseline words). The words included in the pre-test *(baseline* words) and those not included in the pre-test (new words) were thus analyzed separately. Mean *F*_1_ frequencies for the pre-test, test, and post-test are shown in Figures 1, 2, and 3, respectively. Figure 4 shows the average difference in *F*_1_ frequency between the /ε/ and /e/ vowels for each phase (pre-test, test, post-test). For each phase, ANOVAs by participants (*F*_1_) and by items (*F*_2_) were performed with group (shadowing, imitation) and vowel (/e/, /ε/) as variables.

### Pre-test

No significant effect was found (all *p*s > 0.20). Hence, as expected on the basis of our characterization of Southern French, both the shadowing and imitation groups showed no difference in *F*_1_ frequency for the /e/ and /ε/ vowels.

### Test

For both the *baseline* and *new* words, a main effect of the vowel was observed [*baseline*: *F*_1_ (1, 18) = 19.72, *p* < 0.001; *F*_2_(1, 42) = 21.15, *p* < 0.0001, new: *F*_1_(1, 18) = 44.68, *p* < 0.0001; *F*_2_(1, 42) = 36.85, *p* < 0.0001]. Overall, *F*_1_ frequencies were found to be significantly higher for the /ε/ vowel (mean: 506 and 516 Hz for the *baseline* and *new* words, respectively) than for the /e/ vowel (mean: 452 and 449 Hz for the *baseline* and *new* words, respectively). The interaction between groups and vowels was also significant [*baseline*: F_1_(1, 18) = 8.99, *p* < 0.01; *F*_2_(1, 42) = 67.75, *p* < 0.0001, *new*: *F*_1_(1, 18) = 14.39, *p* < 0.01; *F*_2_(1, 42) = 83.07, *p* < 0.0001]. This interaction showed that the difference in *F*_1_ frequency between the /e/ and /ε/ vowels was greater in the imitation than in the shadowing group. The *baseline* and *new* words differed in the decomposition of the interaction. More precisely, planned comparisons indicated that for the *baseline* words, the imitation group exhibited a significant difference between the /e/ and /ε/ vowels but the shadowing group did not [imitation: *F*_1_(1, 18) = 27.67, *p* < 0.0001; *F*_2_(1, 42) = 45.46, *p* < 0.0001, shadowing: *F*_1_ (1, 18) = 1.04, *p* > 0.20; *F*_2_(1, 42) = 2.39, *p* = 0.13]. By contrast, for the *new* words, both groups showed a significant difference between the /e/ and /ε/ vowels [imitation: *F*_1_(1, 18) = 54.88, *p* < 0.0001; *F*_2_(1, 42) = 65.43, *p* < 0.0001, shadowing: *F*_1_(1, 18) = 4.18, *p* = 0.05; *F*_2_(1, 42) = 7.53, *p* < 0.01].

### Post-test

For the *baseline* words, the vowel's main effect was significant by participants but failed to reach significance by items [*F*_1_(1, 18) = 9.28, *p* < 0.01; *F*_2_(1, 42) = 2.63, *p* = 0.11]. For the *new* words, the vowel's main effect was highly significant [*F*_1_(1, 18) = 14.28, *p* < 0.01; *F*_2_(1, 42) = 8.77, *p* < 0.01]. Overall, *F*_1_ frequencies were higher for the /ε/ words (mean: 449 and 458 Hz for the *baseline* and *new* words, respectively) than for the /e/ words (mean: 435 and 432 Hz for the *baseline* and *new* words, respectively). No other effect was significant. Although the interaction between group and vowels was not significant, we tested for the effect of vowel within each group. This was indeed useful since the shadowing group showed no significant *F*_1_ difference between the /e/ and /ε/ vowels on the *baseline* words in the test phase, and thus it would have been surprising if a difference had emerged in the post-test^1^. Again, for the *baseline* words, only the imitation group exhibited a significant difference between the /e/ and /ε/ vowels [imitation: *F*_1_(1, 18) = 9.12, *p* < 0.01; *F*_2_(1, 42) = 4.10, *p* < 0.05, shadowing: *F*_1_(1, 18) = 1.66, *p* > 0.20; *F*_2_(1, 42) = 1.03, *p* > 0.20]. In contrast, for the *new* words, both groups showed a significant difference between the /e/ and /ε/ vowels [imitation: *F*_1_(1, 18) = 10.31, *p* < 0.01; *F*_2_(1, 42) = 10.69, *p* < 0.01, shadowing: *F*_1_(1, 18) = 4.55, *p* < 0.05; *F*_2_(1, 42) = 5.57, *p* < 0.05].

To sum up, convergence effects were observed for participants engaged in an explicit imitation task, but also for participants engaged in a shadowing task in which no explicit instruction of imitation was given to them. The results also showed that imitation instruction led to greater convergence effects than shadowing instruction during the test phase but not during the post-test one. Crucially, additional analyses with group (shadowing, imitation), vowel (/e/, /ε/), and phase (test, post-test) revealed a significant vowel × group × phase interaction for both the *baseline* [*F*_1_(1, 18) = 9.35, *p* < 0.01; *F*_2_(1, 42) = 38.68, *p* < 0.0001] and the *new* words [*F*_1_(1, 18) = 20.29, *p* < 0.001; *F*_2_(1, 42) = 59.74, *p* < 0.0001]. As described previously, this interaction showed that the convergence effect is greater in the imitation than in the shadowing group only during the test phase but not during the post-test one.

## General discussion

In this study, we examined the extent to which the phonetic convergence effect found when participants shadow auditory tokens is an imitation of the speaker. Consistent with previous studies (Goldinger, 1998; Namy et al., 2002; Shockley et al., 2004; Babel, 2010, 2012; Babel and Bulatov, 2012), a convergence effect was found in a shadowing task during the test phase, even though participants were not explicitly instructed to imitate the productions they were exposed to. This convergence effect was smaller in the shadowing relative to the imitation task. Critically, however, the post-exposure effect – especially on the new words – was the same whether the participants were asked to repeat or to explicitly imitate the speaker.

The greater convergence effect with imitation instruction compared to shadowing instruction during the test phase is likely due to attentional factors. Given that participants were asked to imitate the specific pronunciation of the speaker, they have likely paid greater attention to the speaker's indexical features in order to get as close as possible to the specific pronunciations of the words they heard. Nonetheless, when participants' attention was disengaged from the speaker's voice, that is, during the post-test reading task, imitation and shadowing instructions led to the same degree of convergence. It appears thus that the convergence effect found in an imitation task has two major components. The first one seems to be automatic and long-lasting, since the convergence effect is still observed while participants are no longer exposed to the specific pronunciation of the speaker. The second component appears to be dependent on attentional factors and is reflected only during the test phase when participants are exposed to the speaker. In contrast, the convergence effect found in the shadowing task appears to be governed by an automatic long-lasting component which is reflected both when participants are exposed to the speaker and after exposition to the speaker. Hence, the convergence effects found in the imitation and the shadowing tasks seem to share a general mechanism which becomes manifest when we assess post-exposure effects. This mechanism appears to be automatic and taps into the long-term representations of the words in memory. Moreover, the fact that listeners automatically converge toward the speaker in an impoverished social environment, even though there is no obvious reason to do so, reinforces the claim that the convergence effects observed in social interactions may reflect, at least in part, an unintentional process that occurs automatically whenever individuals deal with spoken language (see also Babel, 2010).

In agreement with studies by Babel (2010) and Delvaux and Soquet (2007), we showed that dialectal variation is a key characteristic in the observation of convergence effects. For example, Delvaux and Soquet (2007) provide evidence demonstrating that speakers shift from their dialect to another dialect after a brief period of exposure to the other dialect. Here, we showed that Southern French speakers, who do not produce the /e/-/ε/ contrast in word final position, do so during and after exposure to a Standard French speaker for whom the contrast exists. Interestingly, accommodation to the speaker's accent persisted over a period of time that extended at least to the end of the post-test phase, since participants showed a convergence effect during the post-test reading task even though they were no longer exposed to the speaker's voice. Our results are in line with recent studies showing rapid adaptation to the speaker's accent (e.g., Maye et al., 2008) that persists for a brief period, and thus argue for flexibility in the lexical representations.

This study provides further evidence that detailed traces of spoken words are created during perception. These detailed traces are then used for production, and as demonstrated by Goldinger and Azuma (2004), they appear to be activated during written word recognition. We know from studies on written word recognition that reading involves access to both orthographically- and phonologically-based representations (Coltheart et al., 1979). As a result, reading can also inform us about the nature of phonological representations. In a recent study, Alexander and Nygaard (2008) familiarized participants with two talkers, the first one speaking at a fast rate and the other at a slow rate. After the familiarization phase, participants had to read a text that they were told was written by either the slow or fast talker. The results showed that reading times were slower for participants who thought they were reading a passage written by the slow talker compared to the fast talker. It appears thus that reading involves access to phonological representations that preserve information related to the speaker's voice such as speaking rate, and, in our study, the speaker's accent.

To sum up, clear convergence effects were found in a shadowing task. The convergence effect found in the shadowing task and that found in the imitation task seem to share a general mechanism which is automatic and which taps into the long-term representations of the words in memory. Our results thus argue for the use of fine grained acoustic details during speech perception and production. They also add to the growing body of research showing that detailed phonological information is accessed during reading.

### Conflict of interest statement

The authors declare that the research was conducted in the absence of any commercial or financial relationships that could be construed as a potential conflict of interest.

## Footnotes

^1^As anticipated in the Section“Materials and Methods,”one explanation for the lack of a convergence effect for the shadowing group on the baseline words could be that participants have already pronounced the words in their own accent during the pre-test phase. Although the interaction between vowels and groups was not significant, we nonetheless performed planned comparisons to test for the stability of our results. Indeed, given that the shadowing group on the baseline words showed no convergence effect during the test phase, it would have been rather surprising if a significant difference between the /e/ and /ε/ vowels had emerged during the post-test phase. What happens on the new words during the post-test phase is thus critical in the assessment of post-exposure effects, since it is on these particular words that significant convergence effects were found in both the imitation and the shadowing group, together with a highly significant interaction between groups and vowels.

## Appendix

**Table:** /e/ and /ε/ ending words used in the experiment.

**Baseline words**		**New words**	
**/e/ vowel**	**/ε/ vowel**	**/e/ vowel**	**/ε/ vowel**
Ambré	Bolet	Futé	Gourmet
Hupé	Criquet	Becquée	Muguet
Potée	Forfait	Gaucher	Tiret
Denrée	Sachet	Cuvée	Simplet
Fessée	Rabais	Purée	Bidet
Pépé	Coffret	Rosé	Fleuret
Dragée	Navet	Diarrhée	Toupet
Sablé	Clapet	Flambée	Cornet
Trophée	Livret	Mosquée	Pichet
Dentier	Rouget	Nacré	Boulet
Boisé	Furet	Figuier	Brochet
Journée	Sujet	Année	Mauvais
Papier	Français	Danger	Anglais
Café	Palais	Léger	Jamais
Santé	Aspect	Beauté	Parfait
Été	Après	Pitié	Objet
Degré	Regret	Soirée	Secret
Privé	Juillet	Parler	Complet
Marché	Succès	Quartier	Auprès
Dernier	Respect	Moitié	Projet
Premier	Inquiet	Porter	Concret
Abbé	Progrès	Durée	Abstrait
